# Hospital-Based Healthcare Workers’ Experiences of Involvement in Perinatal Child Protection Processes: A Scoping Literature Review

**DOI:** 10.1177/15248380241247001

**Published:** 2024-04-30

**Authors:** Maegan Johnsen, Melissa O’Donnell, Maria Harries, Colleen Fisher

**Affiliations:** 1 The University of Western Australia, Perth, Australia; 2 The Women and Newborn Health Service, Subiaco, Australia; 3 University of South Australia, Adelaide, Australia

**Keywords:** child protection, hospital, infant removal, interdisciplinary practice, maternity health, pediatric health, social work

## Abstract

As the number of infants entering Out-of-Home Care at birth internationally continues to rise, Hospital-based healthcare workers (HBHCWs) are increasingly likely to become involved in ethically, morally, and legally complex child protection processes. This scoping review aimed to identify and synthesize qualitative literature pertaining to the perspectives of HBHCWs with experiences of involvement in child protection processes occurring in the perinatal period. JBI Methodology for Scoping Reviews guided this review. Databases Ovid MEDLINE, CINAHL Plus, PsycINFO, ProQuest, Web of Science, SCOPUS, and Informit were searched between March 1 and April 30, 2023. Eighteen sources were identified as meeting the criteria for inclusion following screening by two independent reviewers. Data extracted from the included sources are presented in narrative and tabular formats. Involvement in child protection processes is an inherently conflictual experience for HBHCWs and gives rise to internal, interpersonal, and interorganizational tensions. Involvement can have an enduring impact on the HBHCWs, particularly when an infant is removed from hospital by child protection authorities. Appropriate peer, managerial, and organizational level responses are essential to ameliorate risk to HBHCWs themselves and subsequently their practice with women, infants, and families. HBHCWs can provide valuable insight into the challenges of delivering healthcare at the interface of child protection. Future research should focus on building understanding of experiences across disciplines to ensure that interventions designed to prepare and support HBHCWs are effective and evidence-based.

## Introduction

Prior to the 18th century, childbirth was considered a women’s ritual, most often undertaken in the home with the assistance of family members and midwives. With advances in obstetric medicine during the late 19th and early 20th centuries, pregnancy and birth have been transformed. The medicalization of processes occurring in the perinatal period^1^ has subsequently seen pregnant and birthing bodies become increasingly controlled and surveilled within a hospital setting (Sanfelice et al., 2014). Most births around the world are now institutionalized, occurring in a healthcare facility or hospital and attended to by skilled healthcare professionals (World Health Organization, 2023)

Dominant hospital-based discourses, underpinned by medical knowledge about bodily functions and anatomy, have become increasingly prioritized over time and embedded with authority. These dominant discourses have led to the development of medical-models of care, thus positioning medical professionals as the primary experts within a hospital setting, with the expectation that their recommendations are accepted by others, including their patients (Pennington, 2011). However, hospitals represent an intersectional space where medical truths do not exist in isolation, rather these truths exist alongside a diverse and relevant collection of disciplinary perspectives and knowledges, all of which contribute to effective, patient-centered care. Social, legal, and political discourses inform the delivery of perinatal healthcare which, in transitioning from home to hospital, has become increasingly multidisciplinary.

A multidisciplinary approach to the delivery of hospital-based healthcare is known to limit adverse events, improve outcomes, and increase satisfaction for both patients and staff (Everitt et al., 2017). Teamwork is enhanced through a multidisciplinary approach as tensions created by the siloing of knowledge and practice are counteracted and communication and collaborative practice are enhanced (Epstein, 2014). In the case of delivering perinatal healthcare to parents who are at-risk of the removal of their infant at birth by child protection services, trauma-informed, multidisciplinary maternity-healthcare has been shown to reduce stigma, shame, and re-traumatization of this patient cohort who are likely to have experienced adversity and be prone to ongoing psychosocial vulnerabilities (De Backer et al., 2022). Multi-professional communication alongside continuity of care has also been identified as intrinsic to the accurate and consistent evidence gathering to inform child protection decision-making in the perinatal period (Mc Elhinney et al., 2021).

The occurrence of statutory action, where legal guardianship is assumed by child protective services when a newborn infant is removed from its biological parents at or soon after birth, has continued to rise in countries with cognate child protection systems. There was a substantial increase in infant removal rates in Australia at the beginning of the 21st century (O’Donnell et al., 2019), a trend which is replicated in the United Kingdom (Broadhurst et al., 2022; De Backer et al., 2022). Despite being warranted in extreme circumstances to ensure the safety of a vulnerable child, the involuntary removal of a newborn infant from the care of its birth parents (typically a birth mother) by a child protection authority has been described as draconian (Masson & Dickens, 2015), an inherently traumatic event, akin to the experience of still birth or neonatal death but often shrouded in ambiguity, leaving mothers and families vulnerable to experiencing complicated grief (Everitt et al., 2015).

As the number of infants entering Out of Home Care (OoHC) globally continues to rise, so too does the likelihood that healthcare workers employed in maternity and pediatric hospitals will find themselves involved in these ethically, morally, and legally complex processes; processes known to cause professional disquiet (De Backer et al., 2022), secondary trauma and distress, and to have long-lasting emotional impact on the health professionals involved (De Backer et al., 2022; W.Marsh et al., 2020). Hospital-based healthcare workers (HBHCWs) with experiences of involvement in these processes are in a unique position to reflect and advise of the impact on themselves and on the pregnant women, new mothers, and infants to whom they provide services. The aim of this review is to ascertain what is already known about the perspectives of HBHCWs through the multiple disciplinary lenses which exist in a hospital-based healthcare setting.

Attention has been increasingly focused on the risks associated with secondary traumatization of frontline healthcare workers (Arpacioglu et al., 2021). Healthcare workers who deliver care to patients with complex trauma histories pertaining to violence, abuse, substance misuse, and mental health disorders are at high risk of experiencing work-related distress (Fried & Fisher, 2016). Frontline healthcare workers have been identified as being particularly vulnerable to experiencing psychiatric disorders (Lai et al., 2020) with environmental and organizational factors and job characteristics noted as influential on the health and well-being of staff (Gelsema et al., 2005). The extent to which involvement in child protection processes impacts the psychological well-being and ability of HBHCWs to function in a multidisciplinary team must be considered when responding to the needs of all disciplines in maternity or pediatric hospital settings. The effectiveness of this response has the potential to dictate the quality of care being delivered and, thus, influence the outcomes for families.

### Rationale

Despite increased attention being paid to perinatal child protection processes, an initial limited search on MEDLINE and CINAHL Plus identified limited evidence pertaining to the lived experiences of HBHCWs tasked with providing services to pregnant women, new mothers, and infants with child protection involvement. Previous qualitative research has focused primarily on the lived experiences of midwives (Everitt et al., 2015; W.Marsh et al., 2020; Mc Elhinney et al., 2021) and social workers (Dickens et al., 2017; Maslen & Hamilton, 2020; Masson & Dickens, 2015; Mc Elhinney et al., 2021; Nyathi, 2018; J.Thomson, 2016).

A preliminary search of MEDLINE, the Cochrane Database of Systematic Reviews, and Joanna Briggs Institute (JBI) Evidence Synthesis was conducted prior to commencement of this scoping review. No current or underway systematic or scoping reviews on the topic were identified. A systematic review is currently underway at The University of Western Australia which focuses on literature pertaining to the experiences of mothers with child protection involvement in the perinatal period. Concerns about possible duplication have been discussed between authors and while there may be limited cross over of themes there have not been concerns raised about duplication.

Mc Elhinney et al. (2021) completed a systematic narrative review on decision-making by health and social care professionals to protect an unborn baby. A limitation of this review, as identified by the authors, was that the review focused only on midwives and social workers. The current review seeks to expand on the review of Mc Elhinney et al. by capturing literature which attends to the experiences of all HBHCWs who have been involved, either directly or indirectly, with perinatal child protection processes.

## Method

### Study Design

A scoping review methodology was employed in this study as a rigorous and transparent way to identify gaps in existing knowledge in the emerging body of literature which explores the experiences of HBHCWs positioned at the interface of perinatal healthcare and child protection (Munn et al., 2018). This methodology was purposely chosen to ensure that future research on this topic would contribute new and beneficial knowledge to the global body of literature (Khalil et al., 2016) and provide a platform from which to launch future research (Arksey & O’Malley, 2005). This review was undertaken in alignment with the putative framework described by the JBI (Peters et al., 2020). The preferred reporting items for systematic and meta-analysis extensions for scoping reviews (PRISMA-SCR) were employed to depict the identification, analysis, and reporting of data in this review (Tricco et al., 2018)

### Review Question

The question for this review was developed in accordance with JBI Population, Concept, Context (PCC) methodology for scoping reviews (Peters et al., 2021): *What are the perspectives of healthcare workers (Population) on their experiences of involvement in perinatal child protection processes (Concept), including the statutory removal of newborn infants, in a hospital setting? (Context)*

### Review Inclusion Criteria

In alignment with the review question, the inclusion criteria for the review were structured around the JBI PCC framework (Peters et al., 2021). To be included, the source must be peer-reviewed, published in English, have a publication date between January 1, 2000 and February 1, 2023, employ qualitative research methods, include HBHCWs as research participants, and pay specific attention to the perspectives of HBHCWs who have lived experience of involvement in child protection process and focus on hospital-based child protection processes occurring in the perinatal period.

### PCC-Specific Inclusion Criteria

#### Population (P)

*Hospital-Based Healthcare Workers*: Sources were considered eligible for inclusion if the study participants were healthcare workers based in a hospital setting. The review search strategy targeted HBHCWs from both clinical and non-clinical disciplines including medical, midwifery, nursing, psychological medicine, social work, and Aboriginal Health disciplines. Literature which attended to the perspectives of participants who were employed outside of the hospital context were excluded.

#### Concept (C)

1. *Experience of Involvement in Child Protection Processes*: Sources were eligible for inclusion if specific attention was paid in the study to the perspectives of HBHCWs who have lived experience of involvement in non-statutory and/or statutory perinatal child protection processes. A statutory child protection process is one which occurs prior, during or following the removal of an infant from its birth parents’ (most commonly the birth mother’s) legal guardianship by child protective services. Child protection processes which occurred but resulted in the infant remaining in the legal guardianship of its parents are considered non-statutory.

#### Context (C)

1. *Hospital-based*: Sources were considered eligible for inclusion if the study paid attention specifically to the perspectives of healthcare workers employed in a hospital setting who provided services to pregnant women, new parents, and infants under 12 months old with child protection involvement. Literature which focused on the experiences of healthcare workers who provide services outside of a hospital setting, for example community-based child health nurses, was excluded.
2. *The perinatal period*: For the purpose of this review, the perinatal period is defined as the time spanning from conception to 12 months of age ( G.Thomson & Schmied, 2017). The use of this definition was purposeful, as while the primary concern of this review is to attend to the experiences of HBHCWs who provide care during pregnancy, or to infants who have not discharged from hospital following birth, an initial search of the literature identified very limited scholarly work attending to this specific context. The application of a defined perinatal period including a time frame of 12 months postpartum, allowed the authors to capture a broader sample of literature. Literature which attends to child protection processes occurring outside of this defined perinatal period has been excluded.

### Sources of Evidence

Primary, peer-reviewed sources where qualitative methods were employed were considered for inclusion in this review. Mixed methods studies were considered where a qualitative component was present; however, no mixed-methods studies were identified as meeting the inclusion criteria and were thus not included in the review. Relevant data were extracted from each included source. Sources were excluded from this review if deemed to not meet the PCC-specific inclusion criteria, if published before January 1, 2000, in languages other than English and if not peer-reviewed.

### Search Strategy

**Figure 1.**
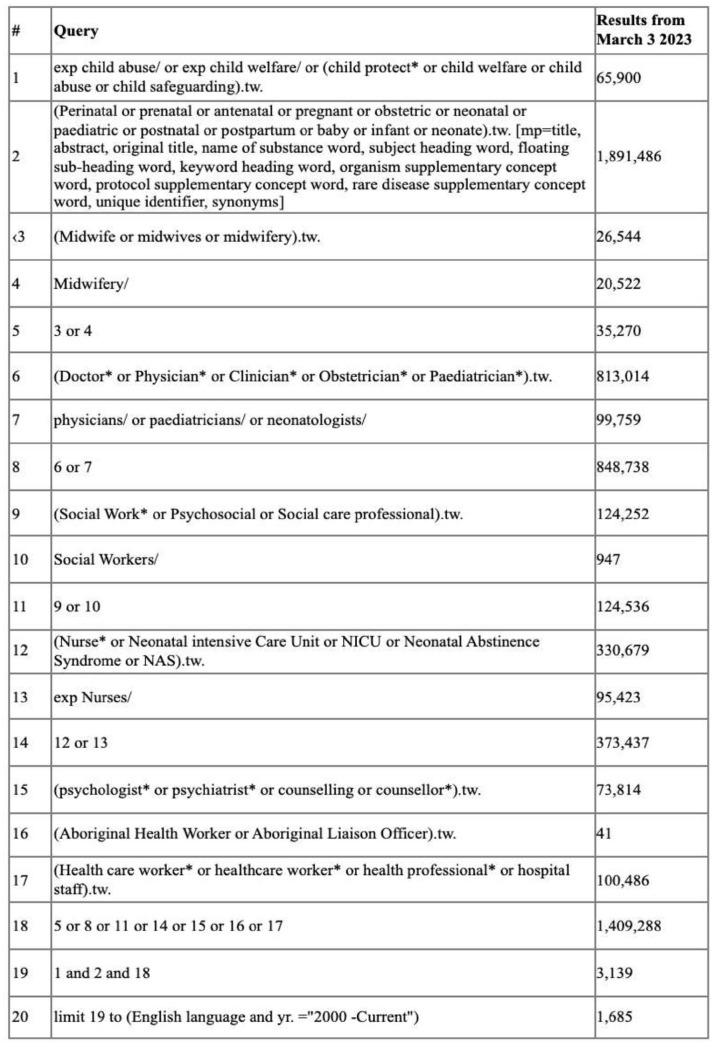
Full search strategy Ovid MEDLINE March 2023.

The JBI framework provided the platform on which the search strategy for this review was developed in order to ensure that studies were identified which would address the research question (Peters et al., 2021). An initial, limited search of MEDLINE and CINAHL Plus was undertaken to pilot the search strategy and identify relevant texts. The keywords contained in the titles and abstracts of these texts and the index terms used to describe them provided the foundation for the final search strategy which was applied across seven databases: Ovid MEDLINE, CINAHL Plus, PsycINFO, ProQuest, Web of Science, SCOPUS, and Informit. The search strategy for this review was adapted to the requirements of each database, although all keywords and index terms were kept consistent across each database searched (see Figure 1 for example of search strategy for Ovid MEDLINE). A citations search was undertaken where the reference lists of all included texts were screened for additional studies.

### Search Outcomes

The principal reviewer undertook a search across seven databases and identified a total of 2,176 sources; all 2,176 sources were uploaded into Endnote 20 and 301 duplicates were removed. A title and abstract review of the remaining 1,875 sources was completed in Endnote 20 with 509 potentially relevant sources identified. Of the 509 sources submitted to a full-text skim, 71 sources were confirmed as relevant and submitted to a further full-text review. Fifteen relevant sources meeting all six inclusion criteria were identified in a full-text review with a further 13 additional sources identified as relevant to the topic but not meeting all six inclusion criteria. A snowball citation sampling undertaken using SPIDERCITE and SCOPUS on the identified sources that met all six inclusion criteria yielded a further 63 potentially relevant sources. The process described above was repeated, and four additional articles were identified for inclusion in the review.

In order to ameliorate the risk of bias, a second independent reviewer completed a title and abstract screening of all sources identified by the first reviewer as potentially eligible for inclusion. A discussion occurred between reviewers when any disagreements or uncertainty arose regarding sources identified for inclusion by principal reviewer. As a result of these discussions, one source initially identified for inclusion by the principal reviewer was excluded as both reviewers agreed the source did not meet all six inclusion criteria. A final 18 sources were identified for inclusion in the review as agreed upon by the principal and second reviewer (Table 1).

**Table 1.** Overview of Study Characteristics and Finding from the Qualitative Literature.

Included Source #	Authors (Year)	Title	Methodology and Design	Participants and Demographics	Extracted Data: Study Aim, Findings, and Conclusions
1	Ahlvik and Lundgren (2022) Sweden	An important task: Midwives’ experiences of identifying children at risk of neglect	Qualitative Phenomenological reflective lifeworld approach	10 Midwives Age range between 26 and 64 years. Experience from 2 to 33 years.	*Aims*: To describe midwives experiences of identifying children at risk of neglect. *Findings*: When working with pregnant or newly postpartum women, midwives hold fears they may misinterpret signals that lead to concerns for neglect. Midwives expressing feel fearful of losing relationships with pregnant or newly postpartum woman if risk is identified and shared. Disagreements reportedly arise with colleagues from different disciplines when midwives’ decisions in relation to risk assessment are questioned. *Conclusions*: Midwives have an important role in identifying children at risk of neglect in the antenatal and postpartum period. When identifying children at risk of neglect, the essential structure of the midwives’ experience is a difficult, complex ambiguous, and divided task.
2	Barrett et al. (2017)Ireland	An exploration of pediatric nurses’ views of caring for infants who have suffered non-accidental injury	Qualitative Framework approach	10 Pediatric nurses Male and female Qualified from 2 to 32 years. Ages range not specified.	*Aim*: To explore pediatric nurses’ views of caring for infants who have suffered non-accidental injury. *Findings*: When providing care to infants with non-accidental injury, nurses report experiencing negative emotions that have lasting effects on their mind and reflections of their own life. Nurses perceive non-accidental injury to an infant as unreasonable compared to other conditions which may lead to hospitalization. Nurses see their care role in this context as part of a legal process and perceive (child protection) social workers as a separate entity who do not valuing the opinions or feedback of nurses. Nurses find speaking to colleagues in similar positions with shared experiences to be helpful and that while peer-support is informal, it is more easily accessible and essential for coping. *Conclusions*: Caring for infants with non-accidental injuries can be a traumatic experience for nurses. Management must pay attention to the experiences of all staff who care for infants suffering from non-accidental injures as the role is challenging and difficult. While peer-support is identified as helpful and accessible, formal support should be discussed openly and without judgment. Training, reflective practice and developing relationships between clinical disciplines would be helpful.
3	Biddle (2019) U.S.	Clinical potpourri for your life and practice	Qualitative Autoethnographic	1 Pediatric nurse anesthetist Author/Participant demographics including age and gender not specified Experience of “more than three decades”	*Aim*: To report the challenges of identifying child abuse. *Findings*: In relation to identifying child abuse, the author encourages clinicians/readers to report any encounter that disturbs or generates concern for the safety of a child. *Conclusion*: The author concludes that to delay action or not act on suspicions of abuse, may lead to missed opportunities to ensure safety of a child.
4	Broadhurst et al. (2022) U.K.	Urgent care proceedings for newborn babies in England and Wales—Time for a fundamental review	Qualitative Thematic analysis	105 Child protection social workers. 46 Forster carers 81 Midwives 22 Support staff from the Children and Family Court Advisory and Support Service 9 Lawyers 44 Parents Participant demographics including age and gender not specified	*Aim*: To report on the findings from the first large-scale qualitative study of professionals and parental experience (*n* = 307) of compulsory state interventions at birth with a specific focus on “urgent care proceedings” *Findings*: Practitioners and parents face multiple challenges when urgent care proceedings are taken. Lack of notice and parental distress in terms of lack of preparation contribute to parents feeling ambushed. Disquiet is salient amongst service providers who are involved in urgent care proceedings for newborn babies. Pressure to discharge postpartum mothers from hospital is compounding the pressure on service providers, including midwives, to work in a transparent and fair way with parents. *Conclusions*: Contemporary hospital systems are not aligned with the needs of vulnerable mothers and families with child protection involvement. Opportunity for extended postnatal hospital admissions could, in many cases, assist in assuaging the need for urgent care proceedings by allowing time for appropriate and fair planning with family when planning was not possible in the pregnancy.
5	Castell and Stenfert Kroese (2016) U.K.	Midwives’ experiences of caring for women with learning disabilities—A qualitative study	Qualitative Phenomenology	9 Midwives Age ranges from 20s to 50s Gender not specified. Experience range from 5 to 10+ years.	*Aim*: To develop an understanding of midwives’ experiences of caring for pregnant women with learning disabilities. *Findings*: Midwives believe that women with learning disabilities are entitled to become parents but there are significant gaps in the health and social services to support them to do so safely. Midwives report feeling as if they are banging their head against a wall as they negotiate time restraints when gaining trust in the context of an inevitable need for a safeguarding role. *Conclusion*: Lack of available support for pregnant women and new mothers and women with learning disability leads midwives to feel unsupported, alone, and holding all the risk.
6	Coupland et al. (2021) Australia	Developing a model of care for substance use in pregnancy and parenting services, Sydney, Australia: Service provider perspectives	Qualitative Grounded theory	38 Hospital-based staff: Managers Social workers Doctors Nurses 39% inpatient hospital focus. 31 Female 7 Male participants. Age range not specified.	*Aim*: To explore service providers’ perception of key components of a model of care for pregnant women or mothers with substance use disorders. *Findings*: Hospital-based perinatal healthcare and community-based healthcare are siloed leading to the lack of continuity of care for pregnant women and new mothers who use substances. *Conclusions*: Inpatient, hospital-based services are limited in the support they can provide to substance using pregnant women, new mothers, and their infants, thus hospital and community-based pregnancy and postpartum care should not sit in isolation of each other. There is evidence to show the benefits of developing a model of care which connects inpatient and outpatient/community services in order to provide better support to at-risk women and families and improve outcomes for those with child protection involvement.
7	Cowley et al. (2018) U.K.	Factors influencing child protection professionals’ decision-making and multidisciplinary collaboration in suspected abusive head trauma cases: A qualitative study	Qualitative Thematic analysis	25 Clinicians (doctors) 10 Child protection social workers. 9 Lawyers 8 Police officers 4 Pathologists Participant demographics including age and gender not specified	*Aim*: to determine factors that influence decision-making and multidisciplinary collaboration in suspected cases of Abusive Head Trauma in infants and children. *Findings*: Diagnosis of abusive head trauma in children is based in knowledge from a wide range of sources. The diagnosis is based on clinical features, the history and social history after other possible causes are eliminated. Providing care to infants with suspected abusive head trauma is emotionally challenging and awareness of bias is essential for clinicians involved in diagnosing and responding when child abuse is suspected. Clinicians find cases of suspected abusive head trauma in infants particularly challenging when the truthfulness of parents as historians must be questions. Barriers exist in decision-making including pressure to diagnose, disagreements between professionals, uncertainty and lack of experience and the potential impact on families. *Conclusion*: Multidisciplinary collaboration and decision-making is usually positive however social workers report being overly reliant on clinical opinions to guide decision-making. Improved training around medical aspects of abusive head trauma for non-clinical professionals and improved understanding between disciplines and agencies is needed.
8	Everitt et al. (2015) Australia	Midwives’ experiences of removal of a newborn baby in New South Wales, Australia: Being in the “head” and “heart” space	Qualitative Thematic analysis	10 Midwives All female Ages ranges from 40 to 59 years old. Experience of between 2 and 30 years.	*Aim*: to explore the experiences of midwives who have been involved in an assumption of care of a baby soon after birth or in the early postnatal period. *Findings*: The authors present two themes; “Being in the Head Space” and “Being in the Heart Space.” The first theme refers to the midwives involvement in or completion of tasks, processes, and activities during the assumption of care. The heart space refers to the emotional impact on the midwife including the perception of how the women they are caring for were treated. *Conclusions*: Midwives feel unprepared and unsupported when involved in the assumption of care process. They connect their experiences of involvement to a professional grief, equating the experience to when supporting a woman whose infant is still born. Recommendations are made for midwives to be supported in order to ensure they can effectively provide care in these complex situations and also attend to the emotional impact on themselves.
9	Everitt et al. (2017) Australia	Working with vulnerable pregnant women who are at risk of having their babies removed by the child protection agency in New South Wales, Australia	Qualitative Thematic analysis	10 Midwives All female Ages ranges from 40 to 59 years old. Experience of between 2 and 30 years	*Aim*: To describe the experiences of midwives who work with vulnerable pregnant women, subject to child protection orders. To address a significant gap in the literature. *Findings*: Effective multidisciplinary teamwork and effective sharing of information will contribute to the best outcomes for vulnerable women and babies. Multidisciplinary teamwork that is cohesive will also support the professionals to continue providing quality care. *Conclusion*: Midwives need to be supported to stay woman centered as keeping the women engaged in antenatal care will also help ensure that the unborn baby remains healthy. Future research is needed to explore the perspectives of all who are involved in the process of a baby’s assumption of care.
10	Itzhaky and Zanbar (2014) Israel	In the front line: The impact of specialist training for hospital physicians in children at risk on their collaboration with social workers	Qualitative Phenomenology	18 Pediatric physicians 14 Social workers 10 Male 22 Female Age range not specified. Experience of 1–20 years (social workers) Physician’s experience level not specified.	*Aim*: To examine the impact of training on cooperation between doctors and social workers to determine if doctors’ increased awareness of the social work role enhanced teamwork. *Findings*: As a result of the training, greater collaboration was evident between doctors and social workers, but friction also occurred between the two disciplines. Increased training for doctors about the role of social work led social workers to feel disturbed by lack of clear boundaries, overlapping of roles which were previously defined and role reversal where doctors shifted focus from medical concerns and began considering psychosocial issues as part of the medical role. Social workers also felt concerned that doctors were too extreme in their reporting and focused only on the child and that child protection reports were made too quickly, were irreversible and often did not take into consideration the nuances of the family’s psychosocial situation. *Conclusions*: Increased, targeted training aimed at reducing differences between doctors and social workers in a hospital setting is recommended as well as adopting a collaborative approach to assessing children at risk. Understanding of another discipline’s role is not enough—teamwork and collaboration are skills that require practice, learning, and standardization.
11	Langer (2011) Germany	A typical Friday	Qualitative Autoethnographic	1 Pediatrician Author/Participant demographics including age and gender not specified	*Aim*: Single author reflection on mistakes made when communicating suspicion of child abuse with parents and colleagues. *Findings*: Advising families of suspicions that child abuse is occurring is an uncomfortable and challenging task. Most doctors prefer comfortable interactions with patients and thus avoid difficult interactions. This avoidance can result in a lack of transparency when providing care to children of families where child abuse is suspected. *Conclusion*: Delaying transparent communication contributes to poor outcomes and can lead to the loss of relationships with families where mutual trust is a fundamental element of the relationship. Training which focuses on having difficult conversations is essential vs. training which only focuses on building good working relationships with families. Maintaining the relationships when having difficult conversations is a skill and key to best practice.
12	Manley (2001) U.S.	An ED nurse reflects on the murders of an infant and toddler.	Qualitative Autoethnographic	1 ED nurse Author/Participant demographics including age and gender not specified	*Aim*: To share experience if caring for abused children that present to pediatric ED and make recommendations for future practice of ED nurses. *Findings*: Caring for children who present to the ED as a result of abuse has a profound impact on staff. Medical professionals can become “enraged” when a decision is made about perpetrators that seem out of alignment with the injuries sustained by the child. Feelings of being insulted are expressed in the context of justifications made for abusive parental behavior. *Conclusions*: ED nurses can have a significant impact on the lives of children who are abused as potentially the first contact who is qualified to identify warning signs of abuse that may be occurring. ED nurses in the U.S. are “mandated reporters” and have a legal obligation to contribute to the health and safety of children. The prevention of child abuse must be prioritized and responses to failures of preventative measures must be immediate and use appropriate legal avenues.
13	C. Marsh et al. (2019) Australia	Making the hidden seen: A narrative analysis of the experiences of Assumption of Care at birth.	Qualitative Narrative enquiry	Mothers Midwives Social workers Child protection workers Exact sample not stated. Participant demographics including age and gender not specified	*Aim*: To study child bearing women and professionals experiences of Assumption of Care at birth. *Findings*: Professionals experience conflicting ethical and moral positions relating to the use/abuse of power, concealment of facts, and “disenfranchised grief.” Increased tension were noted and professionals felt pressure to complete “overlapping roles.” Wider issues associated with the maternity care and child protection systems were noted to exacerbate the unwanted effects of involvement in an Assumption of Care for professionals and mothers. *Conclusions*: Continuity of midwifery care with a known midwife and a therapeutic justice model would assist in ameliorating tensions identified.
14	W. Marsh et al. (2020) U.K.	Removal of babies at birth and the moral distress of midwives.	Qualitative Narrative enquiry with photo elicitation	8 Midwives 4 Mothers Participant demographics not specified.	*Aim*: To explore the experience of moral distress in midwives who provide care to women whose babies are removed at birth. *Findings*: Midwives experience moral distress when providing care to women facing or experiencing the removal of an infant at birth. Caring for women whose babies are removed at birth is reported as one of the most distressing areas of midwifery practice and leaves midwives feeling guilty and helpless. Midwives feel they are betraying the woman/midwife relationship and feelings of distress associated with being the betrayer are experienced for a long time after care ceased to be provided. *Conclusion*: Lack of transparency ruptures the woman-focused care which is the essence of the midwifery role and contributes significantly to the moral distress experienced by midwives when caring for women whose infants are removed at birth.
15	Mc Elhinney et al. (2021) Ireland	Social worker and midwife decision-making regarding child protection risk and the unborn baby: A qualitative study.	Qualitative Thematic analysis	14 Midwives 16 Child protection workers. 24 Female 5 Male Age ranges from 27 to 63+ years.	*Aim*: Explore the perspectives and experiences of midwives and child protection social workers regarding the protection of unborn babies in Northern Ireland. *Findings*: Risk assessment is affected by multiple presenting factors including a pregnant woman’s mental health, drug use, domestic violence, maternal age, feelings about pregnancy, childhood experiences, gestation, and engagement in antenatal care. *Conclusion*: Experience and skill in observation and engagement with pregnant women contributed to professional judgment. Soft intelligence between agencies was important when assessing risk. Continuity of care, multi-professional communication and consistent and accurate evidence gathering were important elements when protecting an unborn baby. Further understanding of models of decision-making in relation to risk assessment is an important area for future research.
16	Saltmarsh and Wilson (2017) New Zealand	Dancing around families: Neonatal nurses and their role in child protection.	Qualitative Grounded theory	10 Neonatal nurses Participant demographics including age and gender not specified Experience of 5+ years	*Aim*: To explore the processes neonatal intensive care nurses used in their child protection role with pre-term infants. *Findings*: Nurses draw on person and professional knowledge to identify the needs and requirements of an infant. Tension occurs when neonatal nurses shape and frame a baby’s safety in the context of the nursing role of protector and nurturer and the baby belonging to its family. *Conclusion*: Involvement in child protection processes is a source of conflict for neonatal nurses and effective child protection work is compromised by lack of confidence in statutory services to respond to concerns raised by nurses. Tension exists for nurses who want good outcomes for infants but who have very limited control over outcomes following discharge.
17	Shannon et al. (2021) Australia	The challenges to promoting attachment for hospitalized infants with NAS	Qualitative Naturalistic enquiry	9 Neonatal nurses and midwives Participant demographics including age and gender not specified.	*Aim*: To explore nurses’ and midwives’ experiences in promoting attachment relationships for infants admitted to an NICU/SCN with NAS. *Findings*: Nurses/midwives value the attachment relationship for infants with NAS however this relationship was mainly promoted when the mother was present in the NICU. Difficulties in promoting the attachment relationship were noted when parents are absent from the nursery or there was child protection involvement. *Conclusions*: Innovative change is needed in regard to promoting attachment relationships for infants with NAS when they are admitted to NICU.
18	Wood (2008) U.K.	Taking the baby away. Removing babies at birth for safeguarding and child protection.	Qualitative Phenomenology	9 Midwives Participant demographics including age and gender not specified Experience defined as from “Band 6—Matron”.	*Aim*: To explore the experiences of midwives in child protection and protecting vulnerable families. Article focuses on the experiences of midwives involved in infant removal at birth. *Findings*: Involvement in the removal of a baby at birth is a highly conflictual and emotive experience for midwives. Some midwives hold on to the memories of difficult experience for years and others cried when discussing poor outcomes and the uncertainty of the future for infants and their families. Midwives often go above and beyond their “call of duty” when caring for pregnant women and new mothers with child protection involvement. *Conclusions*: The safeguarding role of the midwife is “enormous” when protecting unborn and newborn infants. Involvement in the removal of a newborn baby has a significant impact on the midwives involved.

*Note*. ED = emergency department; NICU = neonatal intensive care unit; NAS = neonatal abstinence syndrome; SCN = Special Care Nursery.

**Figure 2.**
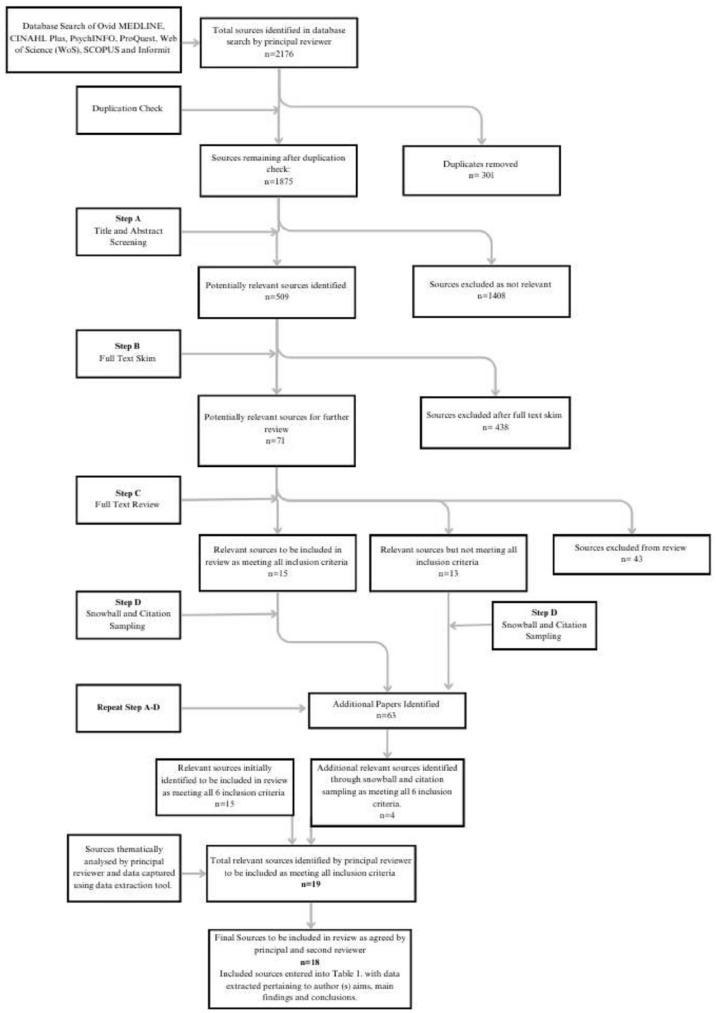
PRISMA flowchart illustrating search process and results. *Note*. PRISMA = preferred reporting items for systematic reviews and meta-analysis.

The review search process and results are illustrated in the PRISMA flowchart (see Figure 2). The 18 included sources originate from countries with cognate child protection systems including 5 from both Australia and United Kingdom, 2 from both United States and Ireland, and 1 from each of Sweden, Canada, Israel, Germany, and Aotearoa New Zealand. Several sources, which initially were identified as possibly relevant following a title and abstract search, were excluded as the full text was published in languages other than English (German and Japanese). In both cases, only the title and abstracts were translated to English and so were picked up in the database search but not eligible for inclusion in the review.

### Data Extraction

**Figure 3.**
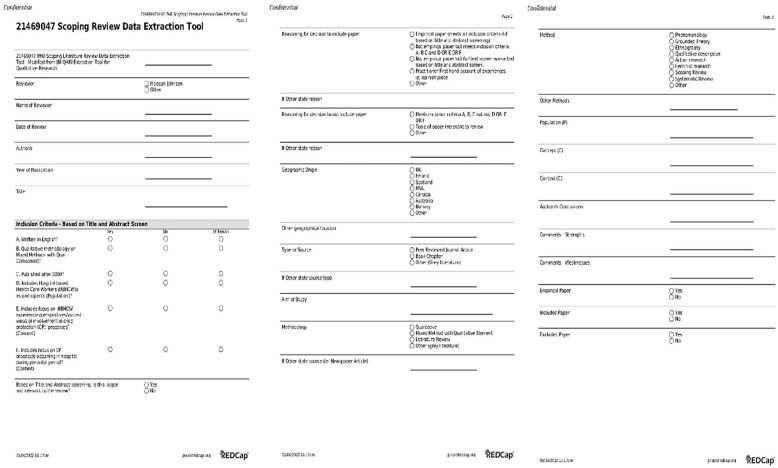
Modified version JBI QARI extraction tool for qualitative research.

A modified version of the *JBI QARI Extraction Tool for Qualitative Research* (see Figure 3) was developed by the first author using the secure web application REDCap. Draft versions of the data extraction tool were piloted and refined during the search process. The first reviewer applied the final data extraction tool to each source identified as meeting the review inclusion criteria to capture fundamental information including authors, publication date, geographic origin, aim, methodology, main findings, conclusions as well as strengths and weaknesses of the source. Extracted data were stored in REDCap for analysis purposes. This information is presented in Table 1.

### Protocol and Registration

As recommended in the JBI guidelines, the protocol for this review has been registered on the Open Science Framework Registry (https://doi.org/10.17605/OSF.IO/CM29H).

### Analysis

A qualitative content analysis using an inductive approach was undertaken on the 18 included sources as per JBI recommendations for scoping reviews (Pollock et al., 2023). Qualitative content analysis is a process designed to synthesize textual data into categories or themes and is recognized as a rigorous method for analyzing documents (Elo & Kyngäs, 2008).

The three phases of conducting a qualitative content analysis as described by Elo and Kyngäs (2008) were adopted in this review: (a) preparation, (b) organizing, and (c) reporting. All authors met regularly during each phase of analysis where processes were discussed, and emerging categories and themes were shared by the primary reviewer (first author) for feedback.

**Figure 4.**
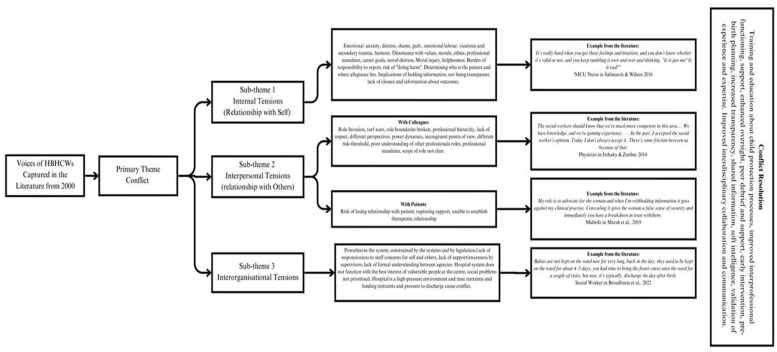
Flowchart showing themes, codes, and examples identified during data analysis.

In the preparation phase, a qualitative analysis using an inductive approach was identified as suitable for this review, given the purpose was to “map” the peer-reviewed literature about a topic on which there is a dearth of evidence (Elo & Kyngäs, 2008; Pollock et al., 2023). The organizing phase involved the first author reading each source several times to comprehend and become familiar with the data (Pollock et al., 2023). To ensure that the relevancy of the data was understood, the review objectives and questions were visible during this process with a printed copy on hand and notes added to digital sources. Once familiar with the data, the first author commenced open coding of the data with codes and reoccurring themes documented until the point of saturation. Inductive qualitative content analysis requires the categorization of codes to be at the discretion of the person undertaking data analysis (Dey, 2003). The reporting phase involved entering the extracted data into tabular form (see Table 1) and the overarching and sub-themes, underlying codes, and examples from the literature which emerged through analysis illustrated in a flowchart (see Figure 4). A narrative summary of the themes which emerged during data analysis was undertaken by the first author and provides a synopsis of the appraised evidence and describes the thematic findings of the review (Pollock et al., 2023).

## Narrative Summary of Themes

### Conflict

Conflict is an overarching theme in the findings of all 18 sources, with three subthemes identified pertaining to the emergence of internal, interpersonal, and interorganizational tensions (see Figure 4). Conflict was a common and overarching experience of HBHCWs required to deliver healthcare while simultaneously being involved, either directly or indirectly, in child protection processes. Conflictual experiences evolve when staff are required to undertake tasks or be involved in processes, which are not congruent with their own values (Manley, 2001), beliefs, or professional mandates (Cowley et al., 2018) especially when the removal of an infant occurs (Wood, 2008). Although statutory removal of an infant at birth is noted to cause the most significant distress in HBHCWs (Everitt et al., 2015; C. Marsh et al., 2019; W.Marsh et al., 2020; Wood, 2008), the child protection processes which occur in pregnancy or in infancy (Saltmarsh & Wilson, 2017; Shannon et al., 2021), particularly those which occur without notice or consultation with the HBHCWs ( W.Marsh et al., 2020), are noted as a catalyst for significant disruption.

### Internal Tensions (Conflict within Self)

Regardless of disciplinary background, delivering healthcare in parallel to child protection planning and decision-making, is an inherently conflictual experience for the HBHCWs involved. Internal, interpersonal, and interorganizational tensions arise through either indirect or direct involvement with the child protection processes at hand, with a higher incidence of psychological distress and trauma reported by those service providers directly responsible for delivering healthcare to women in pregnancy and immediately after birth. A spectrum of internalized tension is evident when service providers describe their experiences from the acute moral distress described by midwives following the removal of an infant at birth ( W.Marsh et al., 2020) to nurses feeling torn between the mandate to protect a vulnerable child and supporting a parent with substance dependency to develop attachment to their infant (Shannon et al., 2021). The salience of professional disquiet is evident across the literature reviewed is noted to be particularly prevalent in cases where staff feel they are unable to fulfill the moral and ethical underpinnings of their professional roles ( C.Marsh et al., 2019).

The process of safeguarding is intrinsic to the professional roles of midwives and nurses (Castell & Stenfert Kroese, 2016; Saltmarsh & Wilson, 2017). However, the burden of this responsibility is reported to be a source of internal stress and confusion. Midwives describe their role as assessors of child protection risk in pregnancy and immediately after birth as ambiguous and complex (Ahlvik & Lundgren, 2022). Midwives report overwhelming feelings of inadequacy and a fear that they will misinterpret the signs of risk with a pregnant woman and subsequently rupture the relationship with her (Ahlvik & Lundgren, 2022). Worries about rupturing relationships with pregnant women and parents and the risk of parental distress, aggression, or disengagement with healthcare can contribute to HBHCWs becoming avoidant of raising concerns about risk (Ahlvik & Lundgren, 2022; Biddle, 2019; Langer, 2011).

It is evident that HBHCWs who are directly involved in care provision prior to, during or immediately after the removal of a newborn infant from a postpartum woman’s care, experience the highest level of distress and secondary trauma (Everitt et al., 2015). Shifting focus from caring for a pregnant woman to protecting an unborn or newborn infant is described by midwives as a struggle and midwives describe acute feelings of anxiety and grief when an infant is removed (Everitt et al., 2017), feelings which are closely linked to the removal being incongruent with a professional mandate which encouraged the notion of partnership where the connection between the mother/infant dyad is prioritized (Everitt et al., 2015). Distress is exacerbated when midwives are required to withhold information from a woman in their care (Everitt et al., 2017; W.Marsh et al., 2020) and midwives report experiencing emotional labor when separating their emotional and the professional selves to ensure continuity of care for women facing the removal of their infants (Everitt et al., 2015).

In the pediatric context, nurses responsible for delivering care to infants suffering from non-accidental injuries in neonatal intensive care units (NICU) and emergency departments report experiencing trauma and a range of strong negative emotions. These negative emotions, including anger, grief, and upset are long lasting (Manley, 2001) and described in the context of delivering care to injured infants while having to continue to support their parents assumed responsible for causing the harm. Nurses report being able to recall traumatic cases from several years prior and throughout their careers and regularly taking work home with them in their mind (Barrett et al., 2017; Manley, 2001). NICU nurses report similar experiences when delivering care to infants with neonatal abstinence syndrome, while attempting to empathize with parents but experiencing internal conflict when they are unable to reconcile the cause of the infant’s illness; maternal substance use (Shannon et al., 2021). Tensions exist for neonatal nurses caring for premature infants with child protection involvement as they oscillate between protecting an at-risk infant to nurturing the bond with the parent to which the infant belongs (Saltmarsh & Wilson, 2017).

### Interpersonal Tension (Conflict with Others)

In addition to tension which arises internally, HBHCWs consistently report the presence of conflict in the workplace between clinicians from different disciplines as well as with the patients they are providing care to. Concerns are raised by midwives regarding disagreements which arise with the questioning of their expertise by colleagues, specifically in terms of risk-assessments and decision-making in a pregnancy. When questioned, midwives report also questioning themselves which affects their sense of professional confidence and decision-making (Ahlvik & Lundgren, 2022). Itzhaky and Zanbar (2014) reported on similar experiences described by social workers when doctors receive child protection training and subsequently become less reliant on those social workers who were previously considered experts. This shift in ownership of expertise leads to social workers experiencing frustration and loss of professional identity within the multidisciplinary team. Despite recommendations for training in hospitals, social workers describe an increase in tensions between disciplines following training designed to improve skills in assessment and response when child protection concerns arise. Itzhaky and Zanbar (2014) found that increasing training for doctors about child protection processes, led social workers to feel disturbed by lack of clear boundaries. Social workers reported the overlapping of roles which were previously defined and role reversal where doctors shifted focus from medical concerns and began considering psychosocial issues as part of the medical role. Social workers also felt concerned that doctors were too extreme in their reporting and focused only on the child, and child protection reports were made too quickly, were irreversible, and often did not take into consideration the nuances of the family’s psychosocial situation (Itzhaky & Zanbar, 2014).

Pediatric nurses responsible for providing care to injured infants in emergency departments raise concerns about disciplinary boundaries, describing how social workers are seen as separate entities who do not value the opinions of nurses (Barrett et al., 2017). This lack of confidence in being believed or respected by other professionals is particularly salient in the NICU environment where nurses provide care to premature infants or infants belonging to at-risk parents (Saltmarsh & Wilson, 2017; Shannon et al., 2021). Midwives interviewed in Ahlvik and Lundgren (2022) describe how the opinions and influence of others can influence how they act, including when in a position of having to assess risk in pregnancy.

In addition to tensions which arise between staff, HBHCWS routinely describe the tensions which occurs in the relationships they have with patients. NICU nurses report feeling frustrated toward absent parents when left providing care for vulnerable infants (Shannon et al., 2021). Fear of losing their relationships with pregnant women is a common theme for midwives (Ahlvik & Lundgren, 2022; Everitt et al., 2015) who share concerns that a woman will disengage from care if aware a midwife is making a report to child protective services. Mutual trust is identified as a fundamental element of productive working relationships. The risk of withholding information from patients when child protection concerns arise is raised as a possible catalyst for loss of trust, leading to poor outcomes (Langer, 2011; W.Marsh et al., 2020). Midwifery is a profession where services are enacted through relational work; concealing information and lack of transparency is connected to the rupture of the “woman-focused” relationships provided by midwives and the woman, especially when there is a plan to remove an infant from a mother’s care immediately following birth and the midwife is not allowed to provide warning ( W.Marsh et al., 2020).

### Interorganizational Tensions (Conflict within and between Organizations)

Conflict arises between HBHCWs and the organizations in which they are situated when the organization is perceived to be unresponsive to the needs of staff and to the needs of vulnerable families they care for. Formalized recognition and support at an organizational level is identified in the studies included in this review, as essential for those working at the interface of hospital-based healthcare and child protection. There is an emphasis on managers being conscious of the challenges experienced by staff responsible for working with injured children and for organizations to respond supportively and without judgment toward those HBHCWs who require or request support (Barrett et al., 2017).

Frustration reported by HBHCWs additionally stems from the wider systemic gaps which lead to inequity and higher likelihood of child protection intervention for women and families with comorbid vulnerabilities (Broadhurst et al., 2022; Castell & Stenfert Kroese, 2016) as well as ongoing racism and discrimination in healthcare settings and the need for more specialist training and support (Everitt et al., 2015; Itzhaky & Zanbar, 2014). HBHCWs interviewed by Broadhurst et al. (2022) specifically correlate the pressure on hospital beds and push to discharge new mothers as increasing the prevalence of infant removal. The lack of alignment between systems and the needs of families leaves staff employed in these systems feeling disenchanted as their ability to deliver the care and support they would otherwise be able to provide (Broadhurst et al., 2022; Castell & Stenfert Kroese, 2016; Coupland et al., 2021). Failure of systems to support families leaves front-end staff holding increasingly levels of responsibility as described by midwives interviewed by Castell and Stenfert Kroese (2016). These midwives express feeling frustrated with the overarching systems in place that do not have the resources to support of young pregnant women with learning disabilities they work with, describing how they feel alone and holding all the risk with no support. Concern is also expressed by HBHCWs who reflect on where the wider, overarching legislative systems fail to protect children from harm (Manley, 2001) and how lack of continuity and support when at-risk mothers transition from hospital to community-based care leaves infants vulnerable to future harm (Coupland et al., 2021).

### Conflict Resolution

The need for improved support for HBHCWs is a prevalent theme across the reviewed literature, regardless of clinical discipline of focus. Support at a peer, line-management, and organizational level is recommended to directly assist those HBHCWs required to care for and support families with child protection involvement (Ahlvik & Lundgren, 2022; Everitt et al., 2015, 2017; Langer, 2011). It is argued that accessing formal support should be normalized and approached with a non-judgment attitude to encourage staff engagement (Barrett et al., 2017). The importance of multidisciplinary collaboration (Cowley et al., 2018; Everitt et al., 2017) and targeted support and training (Itzhaky & Zanbar, 2014) is emphasized to address the concerns raised by HBHCWs with experiences of involvement in perinatal child protection processes. Cross-disciplinary collaboration, understanding, and development at an individual and agency level are put forward as pathways to resolving tensions which have a flow on effect to the care of pregnant women, new mothers, and infants (Barrett et al., 2017)

## Discussion

This review has uncovered limited evidence in the qualitative scholarly literature which pertains to the perspectives and views of HBHCWs with experience of involvement in child protection processes. Perspectives of HBHCWs providing services in the neonatal period before an infant is discharged from hospital following birth were also notably limited. The HBHCWs who participated in the included studies provide valuable insight into the internal tensions which may arise because of involvement in child protection processes. Their reflections elicit insights into the moral and ethical challenges associated with involvement in child protection decision-making and the enduring emotional impact of supporting women and families whose infants are removed at birth (See Table 2 for a Summary of Critical Findings).

**Table 2.** Summary of Critical Findings.

Critical finding 1	The provision of perinatal healthcare at the interface of statutory child protection is an inherently conflictual experience for HBHCWs, an experience which gives rise to internal, interpersonal, and interorganizational tensions.
Critical finding 2	Involvement in child protection processes can have an enduring impact on the HBHCWs involved, especially in cases when statutory action occurs at or soon after birth, where an infant is removed by child protection authorities at a hospital.
Critical finding 3	Addressing the needs of HBHCWs appropriately at a peer, managerial, and organizational level is essential to ameliorate risk to staff well-being and performance.

*Note*. HBHCWs = hospital-based healthcare workers.

What is evident from the included sources is that delivery of healthcare services at the interface of child protection is inherently conflictual, involving tasks that lead HBHCWs to feel conflicted within themselves in their relationships with colleagues and patients and within and between the organizations through which the care is delivered (See Table 2). The medicalization of hospital cultures and implementation of medical-led models of care are seen to compound existing tensions between disciplines where expertise is assumed to be held by those clinicians whose “truths” are prioritized by a medically led system. Child protection work is already ethically, legally, and morally complex, requiring a multidisciplinary and cooperative approach, which can be hampered by tensions related to disciplinary turf wars and perceived hierarchies of expertise.

There is representation in the qualitative literature of voices across disciplinary fields on this topic; however, no evidence of scholarly work which collects, analyzes, and then compares the experience of involvement in child protection processes through the multidisciplinary lenses of the HBHCWs involved. This gap in the literature leaves room for future research which breaks down silos of understanding and knowledge between hospital-based disciplines with the potential to understand the challenges faced at an individual, team, and organizational level (See Table 3 for a Summary of Implications for Practice, Policy, and Research ). Increased knowledge of the dynamics, which exist between HBHCWs from different disciplines employed at the interface of health and child protection, would go a long way toward improving understanding for the betterment of the individuals both receiving and delivering care in this complex space. At an organizational level, benefit would be gained from better understanding the staff experience in order enable them to prepare and respond effectively when conflict occurs or is at risk of occurring (See Table 3).

**Table 3.** Summary of Implications for Practice, Policy, and Research.

Practice	This review has identified how the practice of delivering healthcare at the interface of child protection can be an emotionally, ethically, and morally challenging experience which represents a catalyst for conflict and tension on an individual, interpersonal, and organizational level. This review illustrates how distress associated with involvement can be either exacerbated or ameliorated depending on an individual practitioner’s access to support, preparation and information and overall level of experience. Greater attention is required in terms of responding to the needs of HBHCWs to ensure that practice is safe and sustainable across all levels of experience and regardless of discipline. The existing literature speaks to the value of the lived experience of practitioners and how reflections on experience in the field can directly inform managerial and organizational responses both inside and outside of crisis.
Policy	This review has identified limited evidence in the existing literature of policies which effectively address the needs of HBHCWs who provide care to pregnant women, new mothers, and infants with child protection involvement. It has become evident through this review that HBHCWs can provide valuable insight into the tensions, which arise when delivering services at the interface of hospital-based healthcare and child protection. It would thus be prudent to consider the multidisciplinary perspectives of these HBHCWs when developing future organizational policies designed to respond to the specific needs of staff responsible for delivering care during the perinatal period in a hospital setting. Policies, which are scaffolded on the experiences and specific needs as identified by HBHCWs with lived experience of delivering healthcare at the interface of child protection, will ensure actions are targeted and effective.
Research	This review supports the argument that there is a need for further research to address the gaps in knowledge around the experiences of HBHCWs employed at the interface of perinatal healthcare and child protection. In particular, research which pays attention to the perspectives of service providers from outside of the midwifery, nursing, and social work disciplines which have provided the locus for previous contemporary qualitative studies on this topic. Further scholarly exploration of this topic is required to improve understanding of how involvement in child protection processes can impact the well-being and functioning of HBHCWs at an individual, interdisciplinary, and organizational level and to inform recommendations for practice and policy across multiple disciplines, organizations, and geographic locations.

*Note*. HBHCWs = hospital-based healthcare workers.

## Strengths and Limitations

“Child protection” as a concept is associated with Western, Eurocentric thinking, decision-making, and geographical locations. The purposely restrictive inclusion criteria developed for this review led to the exclusion of potentially valuable sources that could elucidate more culturally diverse perspectives of HBHCWs whose first language is not English. It is important to also recognize that the primary disciplinary backgrounds of participants most often quoted in the included sources (midwifery, nursing, and social work) are female-heavy professions. It is also recognized that participants identifying as female are overrepresented in qualitative research projects where interviews are involved and that female researchers are more likely to undertake qualitative research, thus submitting the data to analysis through a gendered lens which can lead to bias (Plowman & Smith, 2011).

The voices of men and those who identify as male are underrepresented in this review. The limitation of male perspectives in the included sources is further compounded by the exclusion of quantitative and mixed methods sources which used surveys as a data collection tool. It is worth noting that the experiences of doctors on this topic are predominantly captured in the literature using surveys with no open text or comment options. These sources, while valuable in terms of gender representation, did not meet the criteria for this review and thus were not included. Overall, authors of the included sources captured only superficial demographics of research participants, including age, gender, and years of professional experience. A more in depth overview of participant makeup including race, Indigeneity, disability, religion, and lived experience would provide a more nuanced perspective of the participant experiences in question. Having access to this information would also provide the readership with more context through which to understand the perspectives of the participants and how they make meaning of their experiences in this legally, morally, and ethically challenging interface of practice.

Finally, in contemporary politics and media globally, there is much spoken and written about the overrepresentation of Indigenous children in OoHC systems, particularly in Canada and Australia. This review identified a significant gap pertaining to the voices of HBHCWs who identify as Indigenous with no reference to how their Indigeneity may further compound the trauma of bearing witness to the suffering of those with whom they are connected through kinship or culture. This review has not identified scholarly work that includes or explores specifically the perspectives of Indigenous HBHCWs who deliver care at the interface of health and child protection.

## Conclusion

This review has identified significant gaps in the knowledge which exists pertaining to the perspectives of HBHCWs who have been involved in child protection processes, especially those who provide care to new mothers and infants prior to their discharge from hospital. Future research must capture the diverse voices and perspectives of those who are underrepresented in the currently available literature including the voices of male and Indigenous HBHCWs and those who are from disciplines outside of the midwifery, nursing, and social work (See Table 3). The inclusion of detailed participant demographics would also be helpful in future research to provide the audience with more context through which to understand the participant experience and possible bias that occurs as a result of a participant’s background. Research which ensures a diverse range of multidisciplinary voices are contributing to the evidence is essential to inform and improve the support, training, and safety of HBHCWs employed at the interface of maternity and pediatric health and child protection. Appropriate peer, managerial, and organizational level support is essential to help ameliorate risk to the well-being and practice of HBHCWs within the multidisciplinary teams delivering care to pregnant women, new mothers, and infants with child protection involvement (See Table 3).

## Notes

1. For this review, the perinatal period is defined as the time spanning from conception to 12 months of age.
